# Tech-Enhanced Synthesis: Exploring the Synergy between Organic Chemistry and Technology

**DOI:** 10.1021/jacs.5c10303

**Published:** 2025-08-05

**Authors:** Stefano Bonciolini, Antonio Pulcinella, Timothy Noël

**Affiliations:** Flow Chemistry Group, Van ’t Hoff Institute for Molecular Sciences (HIMS), 1234 University of Amsterdam, Science Park 904, 1098 XH Amsterdam, The Netherlands

## Abstract

Recent years have witnessed growing interest in integrating enabling technologies into synthetic organic chemistry to address long-standing challenges in reproducibility, sustainability, and scalability. This perspective showcases how modern tools, ranging from continuous-flow reactors and electrochemical cells to photochemical technologies, biocatalysis, mechanochemistry, and self-driving laboratories, are reshaping the way chemists design, perform, and optimize reactions. Through selected case studies, we highlight how these technologies not only solve specific reactivity and process issues but also open new avenues for reactivity discovery and chemical innovation. Rather than viewing technology as a complication, we advocate for its adoption as a natural extension of synthetic creativity, capable of enhancing safety, reducing waste, and expanding accessible chemical space. Our aim is to inspire broader implementation and interdisciplinary training to equip the next generation of chemists with the tools to rethink how synthesis is performed in the 21st century.

## Graphical abstract

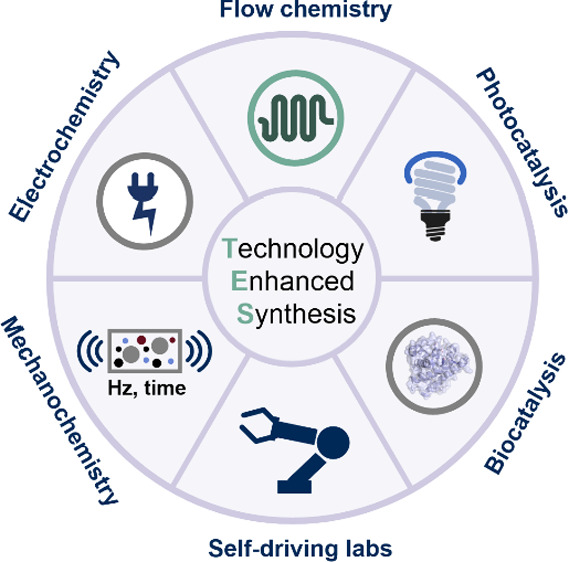

## Introduction

Over the past century, innovations in synthetic organic chemistry have significantly enhanced our ability to develop new drugs, agrochemicals, materials, and other specialty chemicals.^1^ However, the process of crafting the right molecule for a specific application remains time-consuming and complex, and is often compared to an art form.^2,3^ This synthetic bottleneck frequently becomes the limiting step in discovery pipelines, where thousands of molecules must be prepared and screened to fine-tune properties, such as potency, selectivity, and stability.^4^ The challenges associated with this synthetic bottleneck has been addressed by developing new synthetic methods that allow practitioners to rethink or even shortcut synthetic routes. However, the way we set up reactions in both academic and industrial laboratories has remained largely unchanged since the advent of synthetic organic chemistry in the mid19th century.

Most reactions are still carried out in round-bottom flasks, a convention that persists despite the complexity of modern synthetic challenges. As a result, chemists often adapt their reagents and conditions to fit the limitations of the vessel. For instance, hazardous or unstable reagents are often reformulated into more manageable surrogates, and reaction conditions are designed to mitigate risks such as exotherms through dilution or cryogenic temperatures. Yet, alternative technologies, including flow reactors, mechanochemistry, photochemical and electrochemical platforms, and biocatalysis, can handle these challenges more efficiently and safely. Unfortunately, such tools are often adopted only as a last resort, when traditional modifications fail.

One major reason for this technoskepticism^5^ is the lack of training in modern methods within the classical chemistry curriculum. Most practical courses still rely on traditional experiments conducted in round-bottom flasks, without exposure to contemporary technologies. By integrating enabling tools, such as flow systems, electrochemical setups, and photochemical reactors, into both theory and practice, chemists can become more comfortable with innovation and more willing to adopt nontraditional workflows. Another reason for the reluctance is the perceived high cost of purchasing advanced technology. However, this can now be mitigated by the availability of Do-It-Yourself kits,^6^ 3D-printing techniques,^7–9^ and inexpensive electronic toolkits,^10^ which make these technologies affordable and readily implementable. Finally, integrating modern technology requires a multidisciplinary approach, necessitating knowledge of chemistry, chemical engineering, and even programming. Fortunately, excellent primers exist in the literature that can quickly bring researchers up to speed, providing a foundational understanding sufficient to utilize the technology. In our lab, we welcome many visiting MSc and PhD students, and we observe that they rapidly grasp the essence and are able to independently begin working with technologies such as flow chemistry, electrochemical setups, photoreactors or automation. This demonstrates that with proper resources and training, the integration of technology into synthetic organic chemistry is not only feasible but also highly beneficial.

In this perspective, our goal is to highlight the transformative benefits that selected enabling technologies bring to synthetic organic chemistry. For each, we present several illustrative examples that clearly demonstrate their advantages over conventional practices. Our aim is to show that these innovations are not previously overlooked methods, but powerful tools that expand the chemist’s toolkit, enabling improved yields, enhanced selectivity, and access to previously inaccessible reactivities. Moreover, such technologies offer pathways to conduct reactions more efficiently, sustainably, and reproducibly. Technology should not be viewed as a complication, but as an opportunity to make synthetic work more creative, rigorous, and impactful.

## Flow Chemistry

Flow chemistry refers to the process of performing chemical reactions in a continuously flowing stream rather than in traditional round-bottom flasks.^11–13^ Reagents are pumped through channels or tubes where they mix and react under controlled conditions of flow rate, temperature, pressure, and residence/reaction time. This allows for enhanced heat and mass transfer, precise control of reaction parameters, scalability, and improved safety for hazardous or exothermic reactions. It also facilitates inline monitoring and automation (see self-driving laboratories section), enabling rapid reaction optimization.

Flow chemistry is well-suited for challenging transformations, especially those involving gaseous reagents.^14^ In traditional batch reactors, gas–liquid reactions are hindered by phase segregation and by limitations in gas–liquid mass transfer. For instance, the selective conversion of gaseous hydrocarbons into value-added products remains underdeveloped. Methane, the primary component of natural gas, is still predominantly used as a fuel. Its direct functionalization represents a longstanding challenge in synthetic chemistry, due to its high C–H bond dissociation energy.^15^ Achieving bond activation typically requires elevated temperatures and pressures, conditions that are often incompatible with the thermal sensitivity of complex organic molecules. In batch systems, low gas–liquid interfacial areas, limited gas solubility, and safety concerns associated with pressurized reactors further restrict their practical implementation.

Hydrogen atom transfer (HAT) photocatalysis has enabled the homolytic cleavage of C­(sp^3^)–H bonds in gaseous alkanes at ambient temperature, providing access to functionalized alkyl derivatives via radical pathways.^15–22^ To overcome the intrinsic challenges associated with handling gaseous reagents, Noël and co-workers demonstrated the utility of flow reactors equipped with high-pressure capabilities.^14,23^ By combining high-pressure pumps with back-pressure regulators, i.e. valves that maintain system pressure above a set threshold, gaseous alkanes can be effectively liquefied, enhancing their solubility and reactivity. This pressurization strategy not only facilitates the use of gaseous substrates under controlled conditions but also allows precise modulation of reagent stoichiometry and dosing; these are parameters that are difficult to manage in conventional batch reactors where gaseous alkanes are often used in large excess. Consequently, flow systems greatly streamline the practical handling of gaseous reagents, enabling rapid condition screening, reaction optimization, substrate scope exploration, and straightforward scalability.

Using this flow platform, the authors demonstrated the hydroalkylation of electron-deficient olefins, employing tetra-*n*-butylammonium decatungstate (TBADT, (*n*-Bu_4_N)_4_[W_1__0_O_3__2_]) as the photocatalyst under 365 nm irradiation.^24^ The methodology enabled the functionalization of gaseous alkanes (including isobutane, propane, ethane, and methane) with moderate to excellent isolated yields across 38 examples (Figure 1A). The group subsequently reported the photocatalytic carbonylation of gaseous alkanes using carbon monoxide (CO) to access unsymmetrical ketones (Figure 1B).^25^ The use of a microfluidic flow setup allowed for the safe and efficient handling of gas–liquid mixtures, facilitating rapid and scalable incorporation of carbonyl units into inexpensive feedstocks. Notably, even in the unlikely event of a failure, the total volume of CO present within the microreactor system is minimal, equivalent to approximately half the volume of a standard balloon. This inherent limitation on gas inventory mitigates the risk associated with potential leaks or explosions, rendering the handling of toxic gases like CO markedly safer in continuous-flow microreactors compared to traditional batch setups. Such safety enhancements are among the key drivers for the adoption of flow technologies in industry.^26,27^

**Figure 1:**
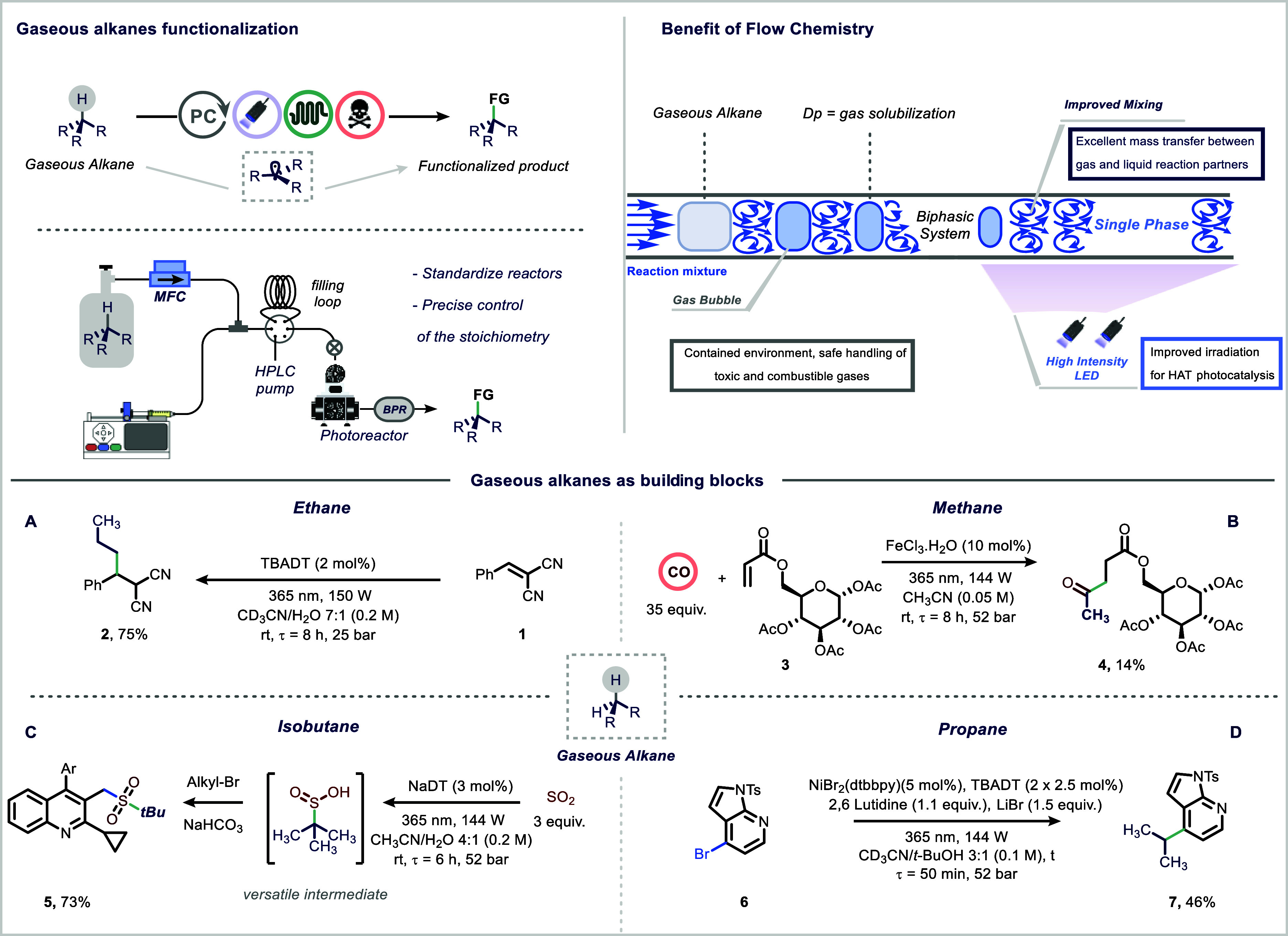
Functionalization of gaseous alkanes in continuous flow.

Next, the authors applied this strategy to synthesize alkyl sulfinic acids by combining gaseous alkanes with sulfur dioxide (SO_2_), a corrosive gas.^28^ The resulting sulfinic acids served as versatile intermediates for the synthesis of alkenyl sulfones, sulfonamides, and sulfonate esters, providing streamlined access to a broad array of sulfur­(II) and sulfur­(IV) compounds (Figure 1C).

In a mechanistically distinct advancement, Noël and co-workers also reported the first cross-coupling of gaseous alkanes with (hetero)­aryl bromides (Figure 1D).^29^ By merging decatungstate-mediated HAT photocatalysis with nickel catalysis, the team enabled direct C­(sp^3^)–C­(sp^2^) bond formation, achieving moderate to good yields across a range of substrates. Recently, a photocatalytic Minisci reaction was successfully applied to install short alkyl fragments (C1–C4) into marketed drugs and natural products.^30^

The transformative impact of flow chemistry becomes evident in the handling of hazardous, toxic, or highly reactive reagents, especially gaseous species. The ability to generate reactive gases on demand within a confined and controlled microfluidic environment circumvents many challenges associated with storage, transport, and accurate dosing.^31^ This is especially advantageous when only small quantities are required, making large gas cylinders impractical or unsafe.

A compelling example is the introduction of the −SO_2_F functional group, which has garnered significant attention in recent years due to its role in sulfur­(VI) fluoride exchange (SuFEx) chemistry, a robust “click” reaction with broad applications in drug discovery,^32,33^ chemical biology,^34^ and materials science.^35^ However, the use of gaseous sulfuryl fluoride (SO_2_F_2_) in SuFEx reactions has been limited by difficulties in safely managing and dosing this toxic gas.^36^ Although crystalline surrogates have been developed, they are often expensive, generate waste, and require SO_2_F_2_ in their own synthesis, ultimately undermining sustainability and atom economy.^37,38^ Therefore, the direct use of SO_2_F_2_ gas remains the most efficient and environmentally favorable strategy.

In response to this challenge, Noël and co-workers reported a modular flow platform that enables the safe, on-demand generation and use of SO_2_F_2_ from inexpensive commodity reagents, specifically sulfuryl chloride (SO_2_Cl_2_) and potassium fluoride (KF) (Figure 2).^39^ The system comprises two interconnected flow reactors: the first is a packed-bed reactor containing KF, where SO_2_F_2_ is generated via a halide exchange reaction; the resulting gas is then immediately directed into a second reactor where it reacts with a chosen nucleophile to form the desired SuFEx product in under 2 min of residence time.

**Figure 2:**
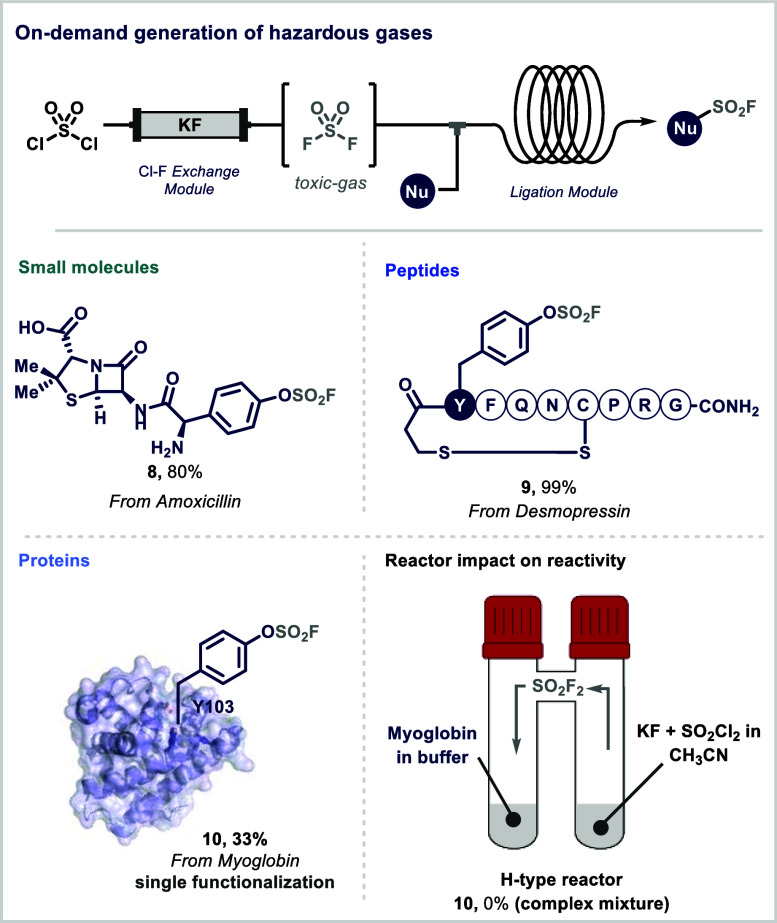
Rapid SuFEx ligation of small molecules, peptides, and proteins in continuous flow.

The utility and versatility of this approach were demonstrated through ligation reactions of small molecules and complex biomolecules. Noteworthy is the efficient functionalization of primary amines, often unreactive under batch conditions. Both natural and synthetic therapeutic peptides were selectively modified at tyrosine residues in good to excellent yields. The fine control over gas stoichiometry afforded by the microfluidic setup enabled site-selective protein functionalization; for example, myoglobin was selectively labeled at Y103 without denaturation or loss of the heme group. In contrast, attempts to perform the same transformation in a H-type batch reactor failed to deliver product, likely due to poor gas–liquid mass transfer and extended reaction times that promote side reactions and decomposition.

In addition to enabling the safe handling of toxic reagents, microreactor technology provides an efficient platform for generating and harnessing sensitive or unstable intermediates.^11,40,41^ A recent example is the modular flow system developed by Noël and co-workers, designed for the streamlined synthesis of heteroatom–CF_3_-containing compounds from readily available nonfluorinated precursors and simple fluoride salts (Figure 3).^42^ Traditional approaches to this transformation typically depend on perfluorinated reagents, which are often costly, atom-inefficient, and environmentally persistent.^43–45^ In light of emerging regulations aimed at restricting the use of polyfluorinated alkyl substances (PFAS), there is growing interest in more sustainable and routes to access heteroatom–CF_3_ motifs.^46^

**Figure 3:**
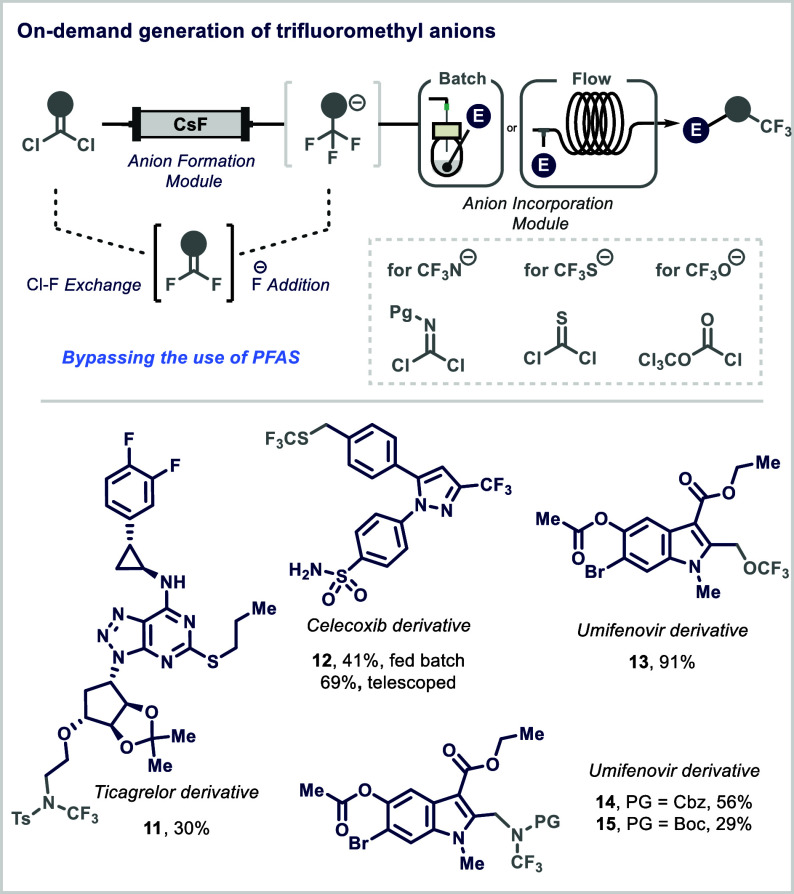
Generation and use of trifluoromethyl-heteroatom anions in continuous flow.

The protocol employs abundant organic precursors and utilizes a cesium fluoride-packed bed reactor to sequentially generate the desired heteroatom–CF_3_ anions.^42^ The process begins with a chlorine–fluorine (Cl–F) exchange, followed by fluoride (F_–_) addition to yield the corresponding anion, which is then reacted with a chosen electrophile. This approach minimizes fluorinated waste and generates only the amount of reactive species needed for the synthesis. Diphosgene, thiophosgene, and protected imidoyl dichlorides were used to access tailored anionic species. The high surface area and efficient mixing within the packed bed enabled the formation of the desired anions within a short residence time of 5–10 min, while effectively containing hazardous gaseous intermediates, such as difluoro­(thio)­carbonyl, preventing their undesired release into the atmosphere. The synthetic utility of the method was demonstrated through incorporation of N-trifluoromethyl­(Protecting Group) [NCF_3_(PG)], SCF_3_ (trifluoromethylthio), and OCF_3_ (trifluoromethoxy) anions into a broad range of electrophiles via a diverse set nucleophilic reactions, such as nucleophilic substitutions and S_N_Ar.

Multistep reaction sequences are fundamental to synthetic organic chemistry, enabling the construction of complex molecules from simple, readily available precursors.^11,47^ However, when performed in batch, these processes are often inefficient, requiring laborious and time-consuming isolation and purification of intermediates. Continuous-flow technology has significantly advanced this area by allowing multiple steps to be integrated into a seamless, uninterrupted workflow.^48–51^ In recent years, there has been a growing interest in adopting this approach for the continuous manufacturing of active pharmaceutical ingredients (APIs).^52,53^ Within this framework, small-volume continuous manufacturing (SVC) has emerged as a promising production model. SVC employs compact, modular equipment operable within standard laboratory fume hoods, yet capable of producing several kilograms of material per day. For example, researchers at Eli Lilly reported the kilogram-scale synthesis of prexasertib monolactate monohydrate (**20**), a kinase 1 inhibitor, achieving a throughput of 3 kg/day for a total of 24 kg of material produced (Figure 4).^54^ Utilizing an SVC integrated with process analytical technology (PAT), the team achieved superior process control, enhanced safety, and effective containment of hazardous intermediates compared to traditional batch processes (Figure 4). The use of low-cost, disposable equipment (e.g., PFA coiled tube reactors) allowed for safe handling of the highly potent prexasertib intermediate without the need for cleaning or cleaning validation, minimizing cross-contamination risks and capital costs. Additionally, the use of identical reactor formats in both development and manufacturing simplified CGMP equipment qualification and streamlined scale-up. The synthesis began with the condensation of α-keto nitrile (**16**) and hydrazine in THF under superheated conditions (130 °C), i.e., temperatures not feasible in batch, which produced amino-pyrazole (**17**) in high yield and purity. This was followed by counter-current extraction and a solvent switch to DMSO using an automated 20 L rotary evaporator. The subsequent S_N_Ar reaction with pyrazine (**18**) yielded intermediate (**19**), which was subjected to continuous crystallization. Final Boc-deprotection, lactate salt formation, and crystallization/filtration steps furnished prexasertib monolactate monohydrate (**20**).

**Figure 4:**
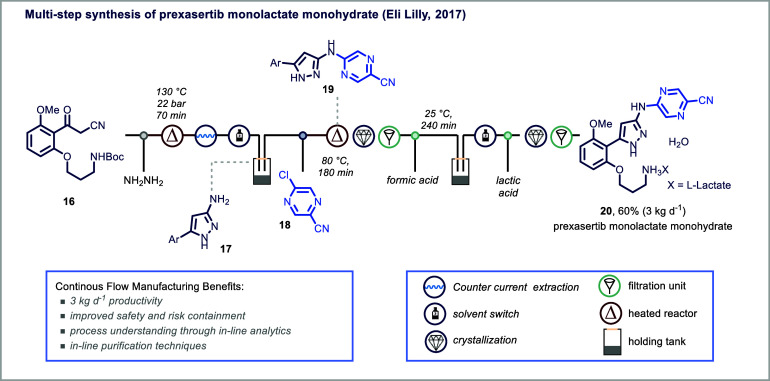
Multistep synthesis in continuous flow.

## Photocatalysis

Photocatalysis has emerged as a transformative strategy in organic synthesis, enabling access to novel reactivity and expanding the scope of chemical transformations.^55–57^ By harnessing light as an energy source, photocatalytic processes facilitate challenging bond activations, particularly via radical-mediated pathways and unconventional cross-coupling reactions.^58^

Technological considerations are central to the success of photocatalysis.^59^ Variables such as reactor geometry, light source, and irradiation intensity profoundly influence reaction outcomes. Yet, key challenges remain, in achieving reproducibility, scalability, and efficient exploration of chemical space.^60^ In this context, the integration of flow chemistry has proven effective for translating photochemical reactions to industrial-scale.^61^ Meanwhile, high-throughput experimentation (HTE) platforms offer powerful tools for rapid reaction optimization and discovery of new reactivity.^62–65^ Equally important is the development of standardized and well-characterized photoreactor systems, which are essential to ensure reproducibility and facilitate broader adoption within the synthetic community.^66,67^

The MacMillan group demonstrated how automated HTE can accelerate the discovery of previously unknown photoredox transformations using a strategy termed “accelerated serendipity” (Figure 5A).^68^ Employing a ChemSpeed robotic platform, they systematically screened combinations of substrates bearing common yet typically unreactive functional groups in 96-well plate format. The reactions were performed under irradiation from a 26 W fluorescent lamp in the presence of an inorganic photoredox catalyst. This unbiased screen led to the identification of a new transformation between N,N-dimethylaniline and 1,4-dicyanobenzene, catalyzed by Ir­(ppy)_2_(dtbbpy)­PF_6_, yielding an α-amino cyanobenzene product in 11% yield. Subsequent optimization of the solvent, base, and catalyst improved the yield to 85%, and the generality of the transformation was demonstrated across a wide range of amines and aryl nitriles. The utility of the methodology was further highlighted by the late-stage arylation of the antibiotic linezolid (**21**). Inspired by this work, numerous groups have since reported mechanistically related transformations.^69–71^

**Figure 5:**
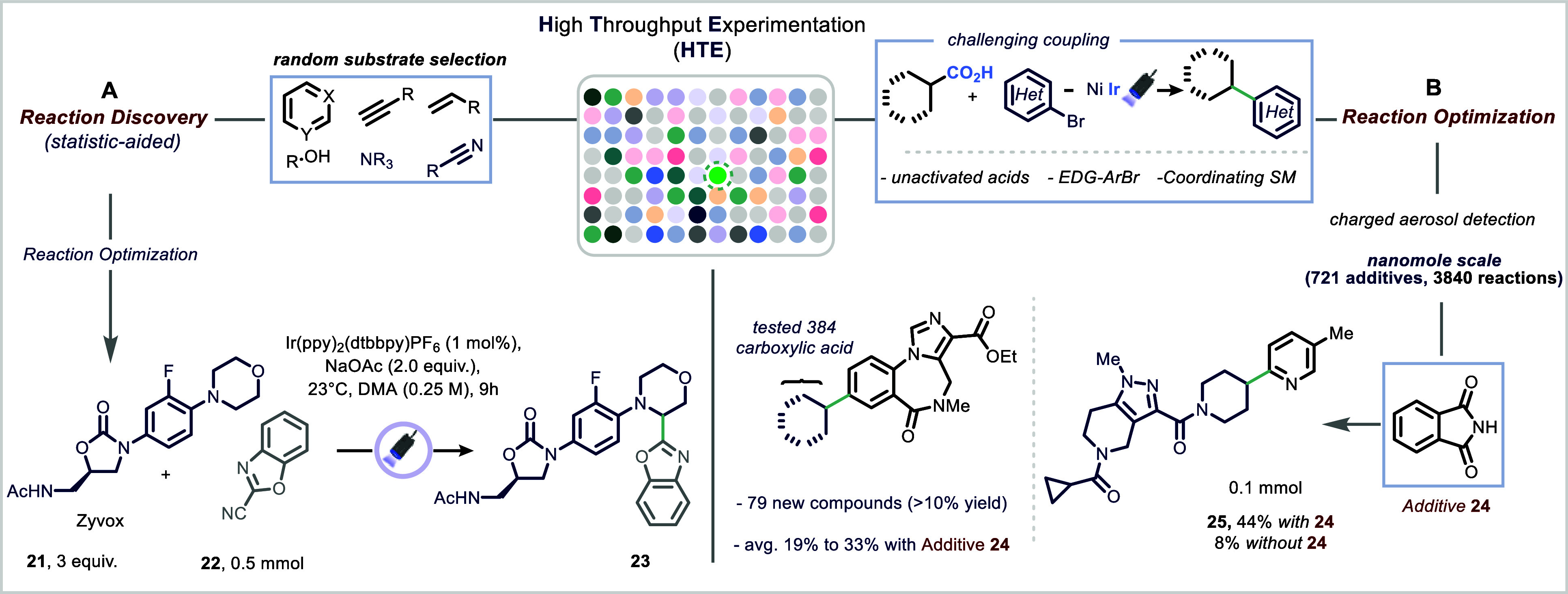
High-Throughput Experimentation (HTE) as enabling technology in photocatalysis.

The MacMillan group introduced an innovative approach aimed at both expanding reaction scope and providing mechanistic insights (Figure 5B).^72^ Drawing inspiration from phenotypic screening in medicinal chemistry, they developed an additive mapping strategy. This technique involves systematic screening of chemical additive libraries to identify modulators that improve reactivity, selectivity, or efficiency, offering a powerful and generalizable framework for reaction discovery and development. The additive mapping strategy was applied to a metallaphotoredox-catalyzed decarboxylative arylation, which is an important transformation that combines photoredox and nickel catalysis to forge C­(sp^2^)–C­(sp^3^) bonds from readily available carboxylic acids and aryl bromides.^57,73^ Despite its synthetic utility, this reaction had previously been limited by narrow substrate scope and suboptimal yields. Through high-throughput screening of 721 organic additives, phthalimide (**24**) was identified as a critical additive that significantly enhanced reaction efficiency. Its inclusion not only improved yields and substrate compatibility but also minimized undesired side reactions such as protodehalogenation. Mechanistic investigations revealed that phthalimide (**24**) helps maintain active nickel species by stabilizing nickel–aryl oxidative addition complexes (OACs) and by reactivating catalytically dormant nickel species.

Despite great interest from the synthetic community, the adoption of photocatalysis in large-scale synthesis, particularly process chemistry, is still in its infancy.^55,74^ According to the Beer–Lambert Law, photon penetration decreases exponentially with depth.^67,75^ Flow chemistry has emerged as a solution by ensuring uniform light exposure across the reaction mixture, offering greater reproducibility.^75^ This enhanced photon flux distribution reduces reaction times, minimizes side-product formation, and improves overall efficiency, making flow-based photochemical processes a viable strategy for large-scale synthesis.

Noël and co-workers developed a scalable decatungstate-photocatalyzed hydrogen atom transfer (HAT) protocol for the conversion of alkanes, ethers, and carbamates into protected hydrazines via reaction with azodicarboxylates (Figure 6A).^76^ The reaction was carried out in a commercial flow photoreactor featuring a perfluoroalkoxy (PFA) capillary irradiated by six chip-on-board UV-A LEDs (totaling 144 W) and actively cooled by an integrated fan.^29,76,77^ Under optimized conditions, the desired protected hydrazine product (**28**) was obtained in high yield. The method was further telescoped to access pyrazoles and phthalazinones without intermediate isolation. Notably, the system achieved a productivity of 314 mmol/h, corresponding to 2.15 kg/day in a single microreactor with just an 11 mL volume (750 μm internal diameter).

**Figure 6:**
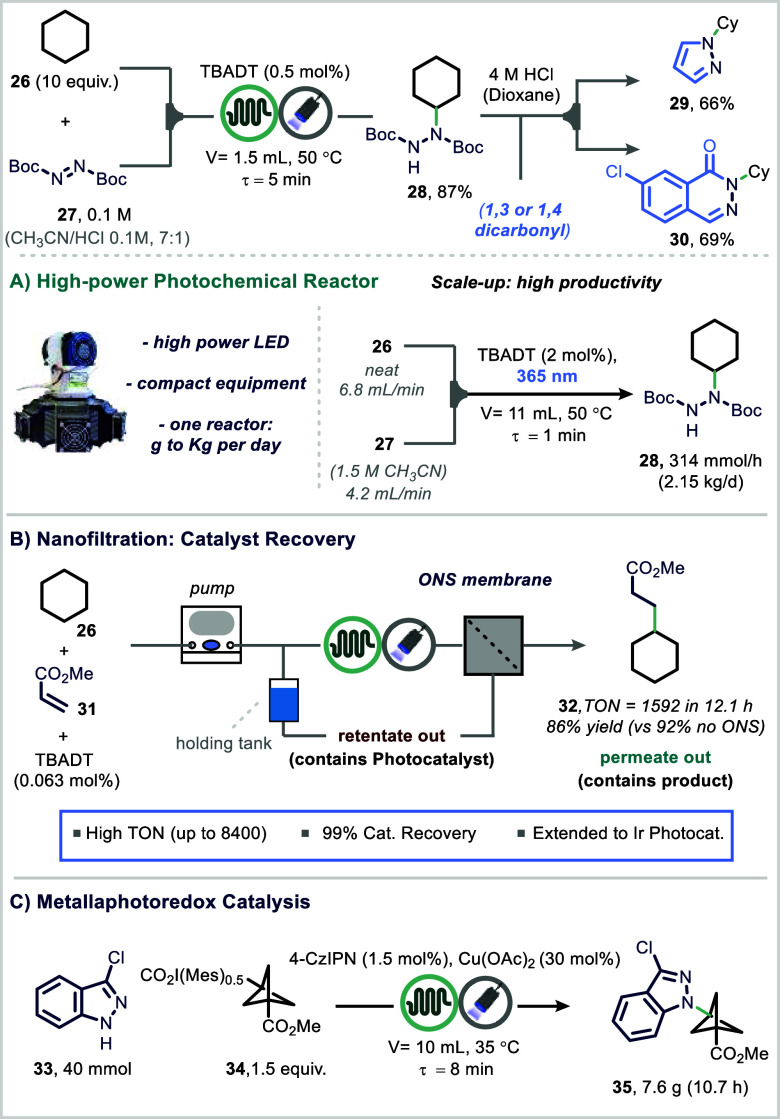
Enabling technologies for the efficient and sustainable scale-up of photocatalysis.

In a follow-up study, Noël and co-workers integrated their microflow photoreactor with in-line organic solvent nanofiltration (OSN) to enable efficient photocatalyst recovery (Figure 6B).^78,79^ A chemically robust commercial membrane (SolSep BV) with a molecular weight cutoff (MWCO) of 500–800 Da was identified as optimal. The setup enabled near-complete catalyst recovery (99%) while preserving filtration performance and catalytic activity, achieving a turnover number (TON) greater than 8400. Moreover, the same group developed an ionic liquid-based version of decatungstate that enabled efficient inline recycling of decatungstate using phase separation.^79^

In a further demonstration of high-throughput experimentation (HTE) in photocatalysis, the MacMillan group developed a platform specifically designed to optimize photoredox reactions for translation into continuous-flow systems (Figure 6C).^80^ By simulating flow reactor conditions at the microscale, this approach bridges the gap between small-scale batch screening and scalable flow chemistry. The study validated this methodology across several representative photoredox transformations, confirming that conditions optimized via HTE could be directly implemented in commercial flow reactors without additional reoptimization. This strategy was exemplified in the decarboxylative coupling of 3-chloroindazole (**33**) and 3-(methoxycarbonyl)­bicyclo[1.1.1]­pentane-1-carboxylic acid derivative (**34**), utilizing a dual copper–iridium photoredox catalytic system.^81^ Reaction parameters were first optimized at microscale using the HTE platform, and the process was then successfully scaled up to a 40 mmol reaction in flow, affording the desired product (**35**) with excellent productivity.

In an industrial context, Merck scientists developed a scalable continuous-flow photochemical bromination of intermediate (**36**), an essential transformation in the synthesis of belzutifan, a recently approved therapeutic for renal cell carcinoma (Figure 7).^82^ Replacing the thermal initiator AIBN with blue light and (**37**), the team achieved a highly selective bromination that suppressed overbromination and degradation pathways. The use of flow chemistry was instrumental in delivering precise control over irradiation intensity and residence time, thereby maximizing selectivity for the desired monobrominated intermediate (**38**).

**Figure 7:**
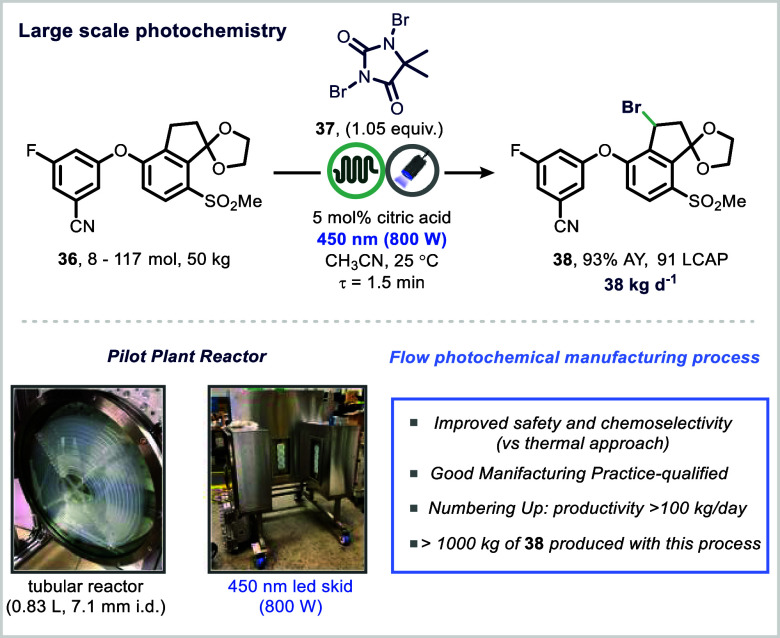
Photochemical manufacturing process in continuous flow. Reproduced from ref 82 with permission from American Chemical Society.

Notably, the study also introduced the concept of “photon equivalents”, which is a parameter quantifying the number of photons delivered per mole of substrate, as a predictive tool for assessing reaction performance across different reactor formats and scales. This metric proved valuable for process development and technology transfer.^83^

The photochemical bromination process was scaled up in three stages. Initially, 3.5 kg of intermediate (**38**) was processed in flow, achieving 88% liquid chromatography area percent (LCAP) and a 94% assay yield with a residence time of 3.75 min. A second scale-up to 50 kg delivered similar results (93% assay yield, 1.5 min residence time). Finally, a numbering-up strategy enabled the production of over 100 kg per day using a GMP-qualified flow reactor, with consistent quality metrics (91% LCAP, 94% assay yield).

## Electrochemistry

Electrochemistry, increasingly recognized as a powerful tool in synthetic organic chemistry, employs electricity as a direct source of electrons to drive diverse chemical transformations.^84–87^ Despite its potential, the broader adoption of electrochemical methods by synthetic chemists was historically limited by challenges such as the complexity of electrochemical parameters, difficulties in reactor design, and a lack of understanding of critical operational variables.^88–90^ However, recent conceptual advances and technological innovations have significantly improved the efficiency, scalability, and accessibility of electrochemical methods, leading to greater integration into mainstream synthetic workflows.

One of the principal challenges in electro-organic synthesis stems from the inherently heterogeneous nature of these processes. Efficient mass transport of reactants to the electrode surface, along with the timely migration of transient radical species between electrodes, is often difficult to achieve.^91^ These limitations frequently result in competing side reactions and degradation pathways. Flow electrochemical cells have emerged as an effective solution, offering a high electrode surface-to-volume ratio and a narrow interelectrode gap.^92–94^ This design minimizes mass transport limitations and reduces ohmic voltage drop, thereby improving reaction outcomes.

A notable development in this field is the microfluidic redox-neutral electrochemical (mRN-eChem) cell reported by Buchwald and co-workers (Figure 8).^95^ This platform features two laser-micromachined glassy carbon electrodes separated by a 25 μm interelectrode gap, maintained by a fluorinated ethylene propylene (FEP) gasket. The small interelectrode distance ensures that molecular diffusion occurs faster than the lifetime of reactive radical intermediates, enabling efficient redox-neutral radical–radical couplings. Using this system, the authors demonstrated a catalyst-free decarboxylative arylation of unactivated carboxylic acids with aryl nitriles.^96^ Mechanistically, anodic Kolbe electrolysis using alternating polarity (AP) generates a transient alkyl radical (**II**), while cathodic reduction of the aryl nitrile (**III**) yields a persistent radical anion (**IV**). This anion migrates to the anode, where it couples with the alkyl radical intermediate; subsequent decyanation furnishes the desired arylated product (**V**) (Figure 8, bottom). When the same transformation was conducted under batch conditions, yields dropped significantly (9%) due to the larger interelectrode distance. Moreover, the device operates without the need for supporting electrolytes, owing to the minimal Ohmic resistance, and is readily scalable to gram quantities, demonstrating its potential for industrially relevant applications.

**Figure 8:**
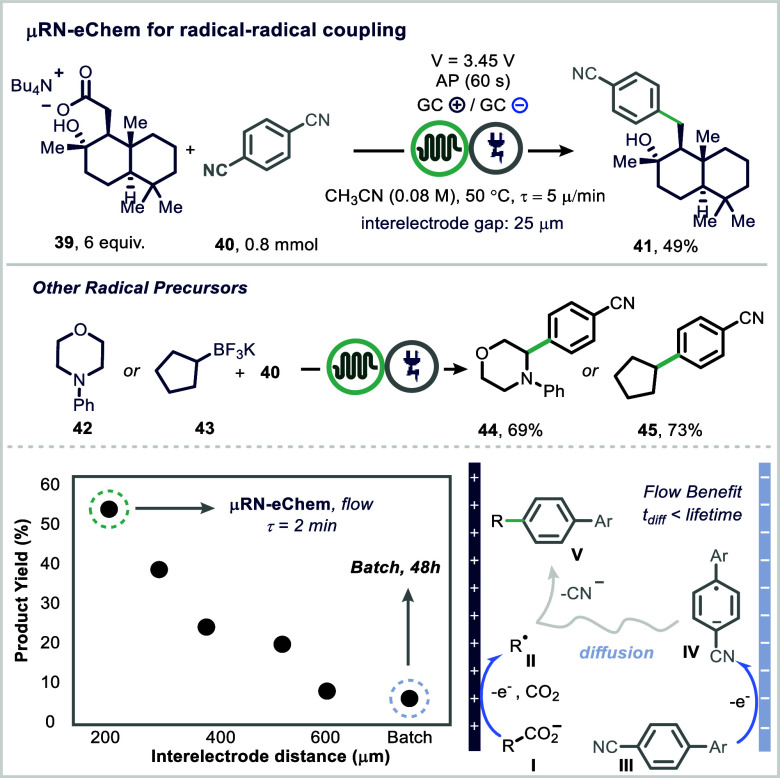
Microfluidic redox-neutral electrochemical (mRN-eChem) platform.

Traditionally, most electrochemical transformations have relied on direct current (DC), where the electrode polarity remains constant and electron flow is unidirectional.^97^ However, recent work by the Baran group has demonstrated the potential of alternating current (AC) to unlock previously inaccessible electrochemical transformations. Despite its widespread use in everyday technologies, from power grids to transportation and electroanalytical devices, AC has rarely been applied in synthetic organic chemistry.^98^ In their approach, the Baran team employed rapid alternating polarity (rAP), a mode in which electrode polarity switches on the millisecond time scale, under either constant current or constant potential conditions. This dynamic reversal suppresses undesired side reactions that occur more slowly than the frequency of polarity change, thus enabling more chemoselective transformations. In an application of rAP, Baran and co-workers achieved the chemoselective reduction of carbonyl compounds, with selectivity outcomes that could be predicted based on substrate redox potentials (Figure 9A).^97^ Notably, this strategy allowed for the late-stage, monodeoxygenation of complex molecules such as proteolysis-targeting chimeras (PROTACs). For example, conjugate (**46**) was only under rAP conditions selectively converted to its deoxygenated analogue (**47**) in synthetically useful yield (75%).

**Figure 9:**
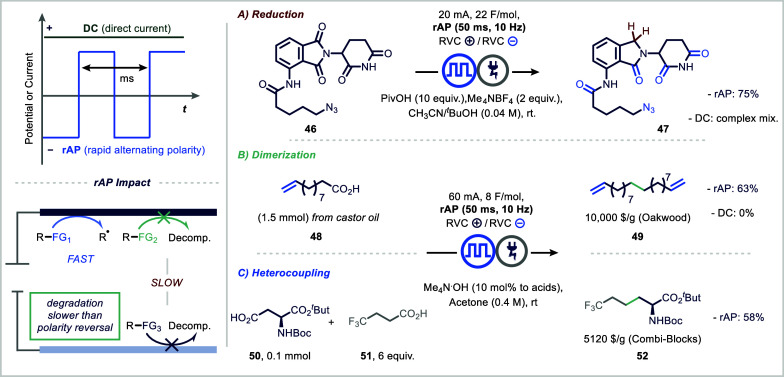
Rapid alternating polarity (rAP) in electro-organic synthesis.

The group more recently extended the rAP methodology to oxidative electrolysis, addressing longstanding limitations associated with the Kolbe reaction. While the Kolbe electrolysis represents a valuable strategy for constructing C­(sp^3^)–C­(sp^3^) bonds in a metal-free fashion, its broader application has historically been hindered by poor functional group tolerance and limited scalability (Figure 9B,C).^99^ Despite its conceptual appeal, the development of general, metal-free methods to forge C­(sp^3^)–C­(sp^3^) linkages remains a notoriously underdeveloped area in synthetic organic chemistry.^100–102^ The implementation of rAP in this context offers a promising solution, enabling selective oxidation of carboxylates while suppressing competing side reactions, and thereby expanding the practical utility of Kolbe-type bond formations under milder and more scalable conditions. Using inexpensive reticulated vitreous carbon (RVC) electrodes, technical-grade acetone as solvent, and catalytic ammonium hydroxide, a range of native carboxylic acids were efficiently transformed into value-added products. For instance, homodimerization of biomass-derived 10-undecenoic acid (**48**) yielded diene (**49**), an industrially valuable yet costly polymer precursor. Similarly, the method enabled the synthesis of complex unnatural and dimeric amino acids from readily available natural amino acids, through either heterocoupling or decarboxylative radical–radical dimerization (**52**).

Mechanistically, a key advantage of the rAP approach lies in its modulation of local acidity at the electrode surface. In conventional DC electrolysis, anodic processes often generate localized acidic environments, leading to protonation of carboxylate groups and subsequent undesired oxidation of other functionalities. In contrast, the rapid polarity switching of rAP prevents the sustained buildup of acidity at the anode, enabling the selective oxidation of deprotonated carboxylates and thereby enhancing both yield and functional group tolerance.^88,99^

As the complexity of electrochemical transformations grows, so too does the need for tools that enable rapid and systematic screening of conditions.^103,104^ To address this, the Lin group drew inspiration from microelectronics and developed SPECS (Small Photoelectronics for Electrochemical Synthesis), which are microfabricated photoelectronic devices that enable high-throughput electrochemical experimentation (HTE) (Figure 10).^105^ These devices, powered by visible light, are manufactured using standard nanofabrication techniques and operate wirelessly at the microliter scale.^106^ When integrated into conventional 96- and 384-well microtiter plates, SPECS effectively transform them into miniature electrochemical reactors. SPECS function through arrays of miniature silicon photodiodes that, under light irradiation, generate electrical potential between integrated electrodes. The magnitude of the current is directly proportional to light intensity and photodiode size, allowing for precise control over reaction conditions. The streamlined fabrication process permits the production of over 1500 devices on a single 4-in. wafer, offering significant cost efficiency, scalability, and ease of reuse.

**Figure 10:**
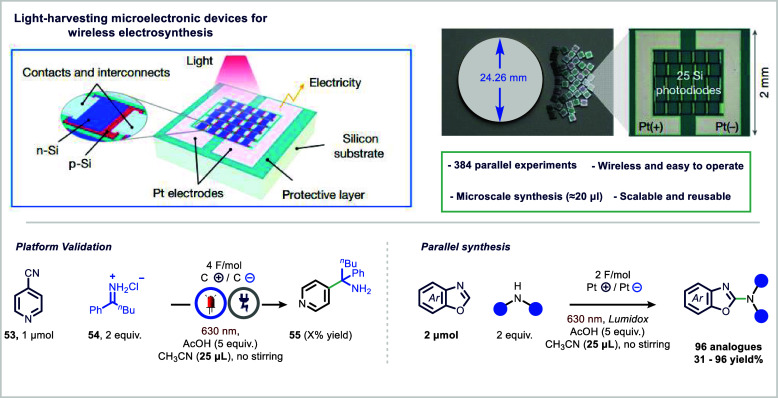
Light-harvesting microelectronic devices for wireless electrosynthesis. Reproduced from ref 105 with permission from Springer Nature.

The utility of SPECS was validated through successful replication of various known electrochemical reactions, including oxidative, reductive, and paired electrolysis pathways. Furthermore, their effectiveness was demonstrated in library synthesis efforts, such as arene C–H amination and the discovery of a novel one-step aza-Shono coupling.^107^ The devices delivered high reproducibility, tunable scalability, and compatibility with diverse electrode materials (e.g., platinum, carbon).

## ‘Self-Driving’ Laboratories and Machine Learning

The integration of automation, machine learning, and robotics into experimental workflows has given rise to self-driving laboratories (SDLs), which constitute a transformative paradigm in modern chemical research.^108–110^ These autonomous platforms operate through the continuous feedback loop of hypothesis generation, experimental execution, data acquisition, and iterative optimization, effectively mimicking the scientific method in silico and in hardware. By coupling AI-driven decision-making with robotic execution and real-time analytics, SDLs enable chemists to navigate vast chemical spaces more efficiently and reproducibly than ever before.

Traditionally, the optimization of chemical reactions or material properties requires extensive manual experimentation, often guided by intuition, incomplete data, or trial-and-error approaches. In contrast, SDLs are designed to autonomously plan, run, and interpret experiments, accelerating discovery timelines while reducing human bias, labor intensity, and resource consumption. These platforms have already demonstrated substantial impact in areas such as reaction optimization, formulation development, catalyst screening, and drug discovery, where high-dimensional parameter spaces and complex data sets challenge conventional workflows.

Despite their promise, SDLs remain in the early stages of adoption within synthetic organic chemistry. Key barriers include the complexity of integrating hardware and software components, the need for robust data infrastructure, and the challenge of encoding chemical intuition into machine-readable formats. Nevertheless, recent advances in modular robotics, closed-loop machine learning algorithms, and open-source platforms have significantly lowered the barrier to entry. As SDLs become more accessible and user-friendly, they are poised to redefine how chemists approach experimentation, thus transforming iterative benchwork into a data-driven, autonomous process.^111^

A demonstration of this potential was recently reported by Noël and co-workers, who developed RoboChem, a multipurpose robotic platform for the autonomous optimization and intensification of photocatalytic transformations.^112^ The system combines modular, off-the-shelf hardware, including syringe pumps, a liquid handler, and an inline benchtop NMR analyzer, with a high-intensity capillary photoreactor and a custom software suite (Figure 11). Central to RoboChem is a Bayesian optimization algorithm that autonomously explores the reaction parameter space, including light intensity, catalyst loading, residence/reaction time, and concentration, to identify optimal conditions tailored to each substrate.^113–115^ The use of flow chemistry ensures reproducibility across experiments, addressing common challenges in photocatalysis such as inconsistent photon, mass, and heat transfer.^59^ The platform was applied to a broad range of light-driven reactions, including hydrogen atom transfer (HAT), photoredox catalysis, and metallaphotocatalysis, and proved particularly effective at refining reaction conditions for complex or sensitive substrates. In a representative example, RoboChem successfully differentiated between the optimization needs of Sclareolide and Ambroxide during a trifluoromethylthiolation reaction.^116^ While Sclareolide required higher catalyst loading and light intensity, Ambroxide’s propensity for overfunctionalization necessitated a gentler approach (Figure 11).^16^ The algorithm autonomously identified these distinctions, improving yields and selectivity for the monofunctionalized product, an outcome that would likely be overlooked in a manually designed scope study, where generic conditions are often applied uniformly across all substrates.^117^ This ability to generate substrate-specific data sets not only improves synthetic outcomes but also provides valuable insights into the interplay between structure and reactivity. Moreover, the high-intensity photoreactor enabled seamless scale-up from milligram to gram quantities within the same setup, bridging discovery and production in a single, reproducible environment. Importantly, the RoboChem platform requires no specialized expertise in photocatalysis or automation, making it accessible to nonexperts and highly attractive for deployment in both academic and industrial laboratories.

**Figure 11:**
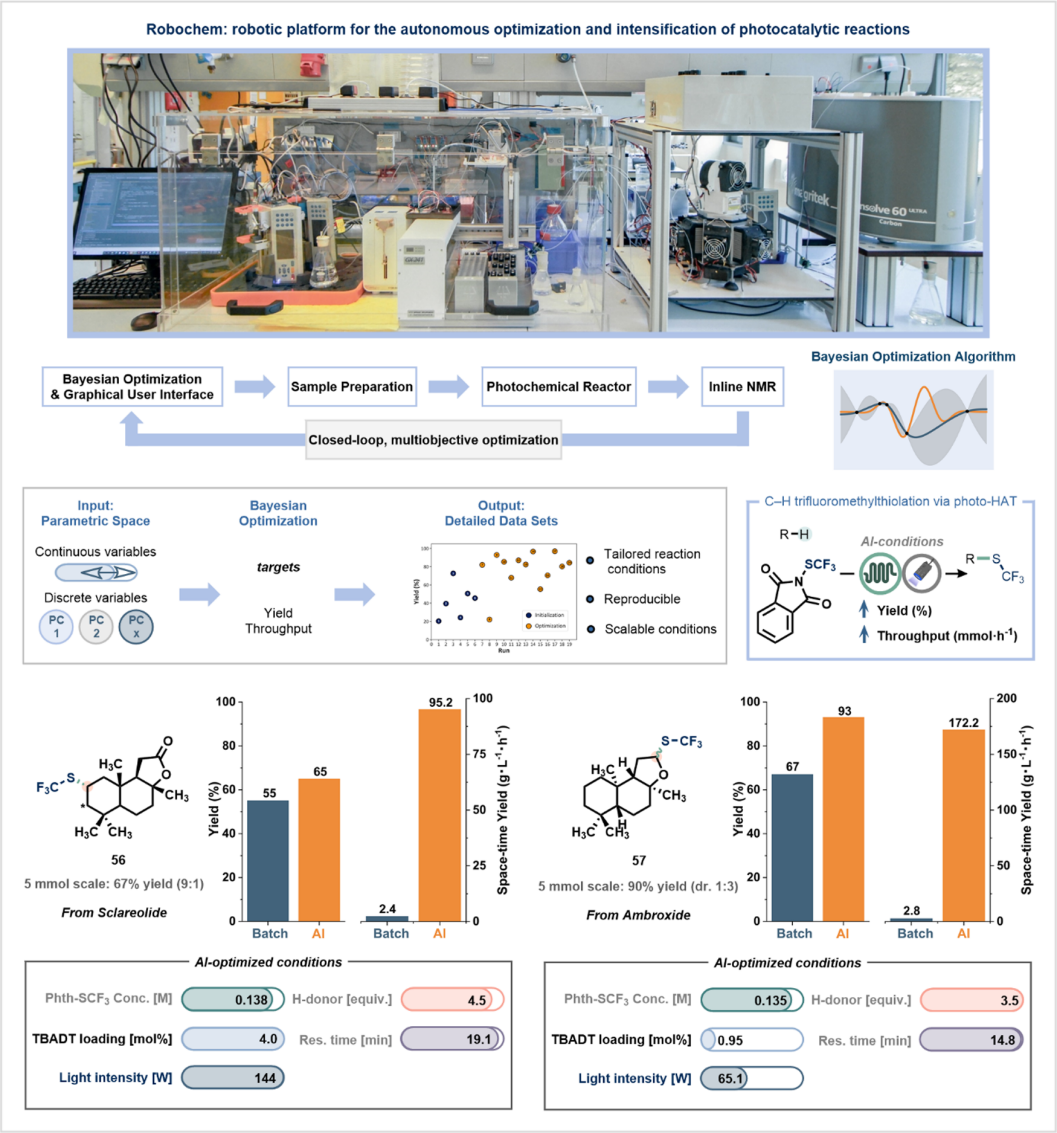
RoboChem: multipurpose robotic platform for the autonomous optimization and intensification of photocatalytic reactions. Reproduced from ref 112 with permission from The American Association for the Advancement of Science.

Beyond single-lab automation, SDLs are increasingly being developed as distributed platforms, where multiple robotic systems across different geographic locations collaborate toward a unified research goal. In a landmark example, a network of SDLs coordinated by a cloud-based experiment planning system was used to discover new organic semiconductor laser (OSL) materials (Figure 12A).^118^ The project employed a modular building-block strategy, combining iterative Suzuki–Miyaura couplings with a generalizable two-step, one-pot assembly of pentameric gain materials.^119,120^ Spanning a virtual library of over 150,000 candidate structures, the synthesis and testing workflows were distributed across four sites, each equipped with robotic synthesis platforms capable of executing tasks assigned by a central machine learning planner. The AI module, enriched with insights from quantum chemical simulations and constrained by real-time feedback on synthetic feasibility and resource availability, continuously reprioritized experiments to maximize learning efficiency.

**Figure 12:**
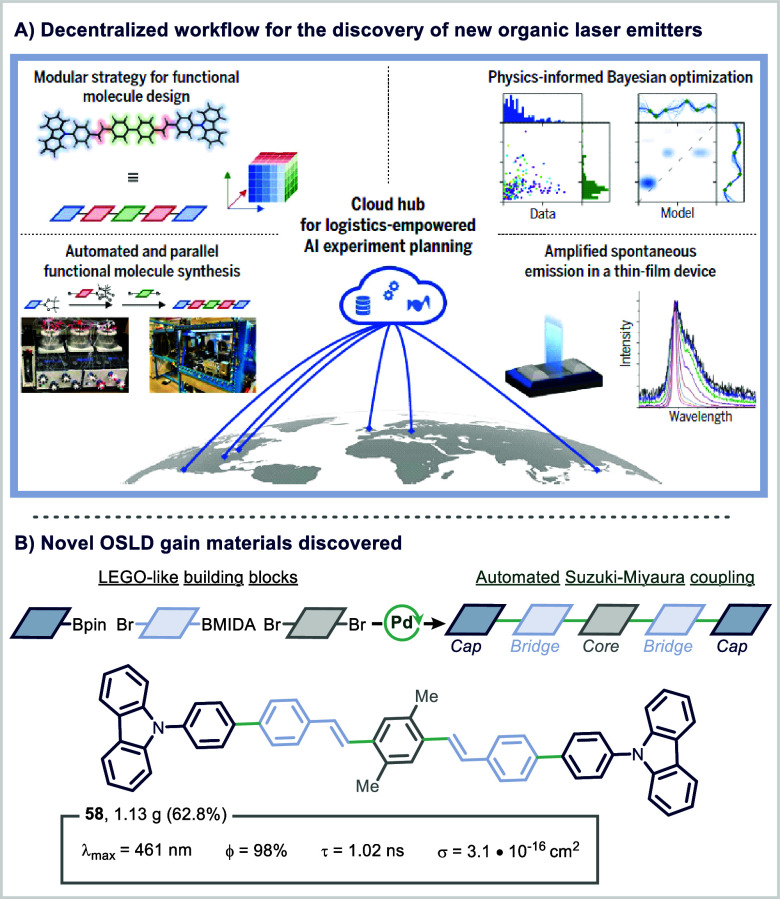
Network of SDLs coordinated by a cloud hub for new OSL materials. Reproduced from ref 118 with permission from The American Association for the Advancement of Science.

This collaborative SDL network ultimately identified 21 new high-performance OSL materials, three of which exhibited best-in-class amplified spontaneous emission (ASE) thresholds in thin-film devices (Figure 12B). To validate these discoveries, the system implemented automated workflows for scale-up, purification, and device-level evaluation. Notably, the integration of physical and logistical constraints into the cloud-based planner ensured seamless orchestration of tasks across geographically dispersed laboratories. This asynchronous, multisite research engine provides a compelling model for future SDL frameworks, where expertise, equipment, and data can be synergistically leveraged across institutional boundaries without geographical limitations.

A third and conceptually distinct example of data-driven application lies in the domain of chemical waste valorization.^121^ The Allchemy platform represents a computer-aided approach to reaction planning that applies forward-synthesis algorithms to identify viable synthetic routes from industrial waste chemicals to high-value targets (Figure 13A). By mapping tens of thousands of reaction pathways from ∼200 waste substrates, many of which are commercially abundant or environmentally burdensome, Allchemy generated a vast synthetic network encompassing routes to over 300 known pharmaceuticals and agrochemicals. These pathways were ranked algorithmically using metrics of sustainable chemistry, such as process mass intensity (PMI), *E*-factor, and synthetic convergence (Figure 13B).

**Figure 13:**
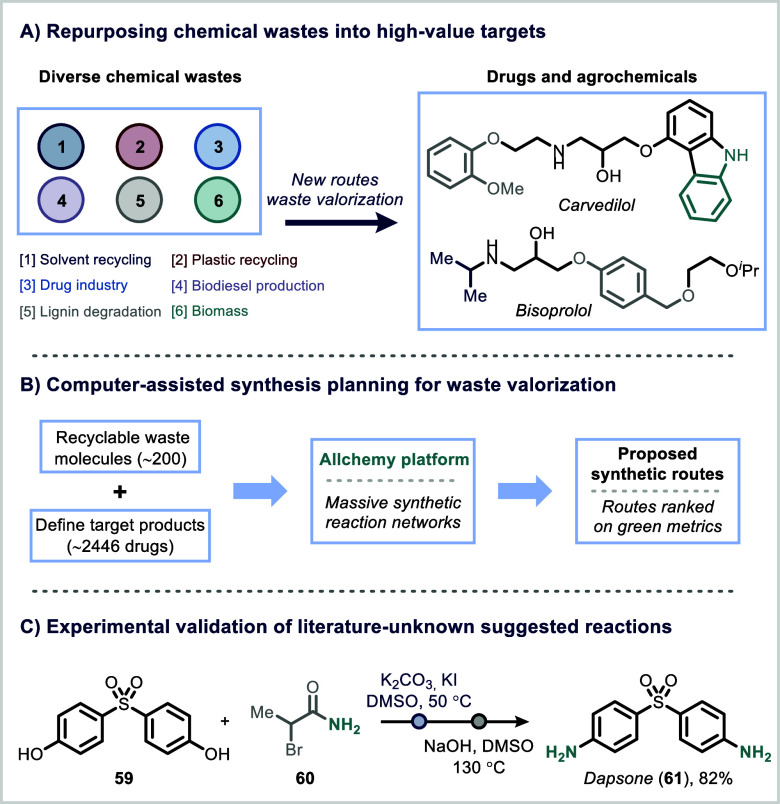
Repurposing chemical waste via computer-aided synthesis planning.

To assess the practical feasibility of the top-ranked routes, several transformations were experimentally validated, including the synthesis of the antibiotic Dapsone using feedstocks derived from waste streams such as plastic recycling (lactic acid) and lignin degradation (phenol, Figure 13C). The ability to generate and evaluate entire synthetic networks algorithmically, while incorporating both green chemistry metrics and real-world constraints, demonstrates the utility of computer-aided synthesis planning (CASP)^122,123^ in addressing large-scale sustainability challenges. This model also suggests a potential industry-wide platform, where different stakeholders, such as chemical manufacturers, waste managers, pharmaceutical firms, could input desired outputs or available substrates, and SDL-guided systems would propose and coordinate the necessary synthetic efforts. Such a system would enable digitally driven circular chemistry at scale, contingent on wider adoption and regulatory incentivization.

Together, these case studies underscore the expanding scope of self-driving laboratories and data-driven approaches in modern synthetic chemistry. Whether deployed to optimize reactions, accelerate discovery, or reimagine waste-to-value pathways, SDLs and CASP offer a fundamentally new approach to experimentation, one that is autonomous, data-rich, and scalable. As automation technologies continue to mature, and as open-source platforms and modular components become increasingly accessible, SDLs are poised to become integral tools in both academic and industrial settings.

## Biocatalysis

Biocatalysis plays a central role in sustainable pharmaceutical manufacturing, owing to the unique advantages of enzymatic transformations.^124–126^ These include the use of water as a reaction medium, high substrate specificity, exceptional regio- and stereoselectivity, and the inherently renewable and biodegradable nature of enzymes. Enzymatic processes are generally safe and energy-efficient, making them particularly attractive for green chemistry applications. However, the broader application of natural enzymes is often restricted by their narrow substrate scope and reduced catalytic performance outside their native biological environments. These limitations present major challenges for large-scale implementation, where robust performance under diverse and demanding conditions is essential.^127^

To overcome these barriers, directed evolution (DE) has emerged as a powerful technology, allowing the systematic modification of enzymes to improve activity, broaden substrate tolerance, and enhance stability under process-relevant conditions.^128–130^ Through iterative cycles of mutagenesis and selection, DE has made it possible to tailor enzyme function to meet the demands of industrial synthesis.^127^

This section highlights practical applications of engineered enzymes in pharmaceutical settings, demonstrating how DE has been used to streamline access to clinical drug candidates and valuable intermediates.^131–134^ Continued innovation in the field is expected to be driven by advances in computational enzyme design,^135,136^ machine learning for predictive structure–activity modeling,^137^ ultrahigh-throughput screening,^64,138^ genetic code expansion,^139,140^ and the incorporation of novel catalytic motifs.^141,142^ Together, these emerging technologies are accelerating the development of artificial enzymes, bridging the gap between nature-inspired catalysis and conventional synthetic methods.^143^

A seminal example of this approach was reported in 2019 by Merck and Codexis, who developed a fully in vitro, nine-enzyme cascade for the synthesis of islatravir (**66**), a nucleoside analogue under investigation for the treatment of HIV (Figure 14).^144^ Starting from a simple, achiral building block (compound **62**), the cascade delivered high stereocontrol and minimal waste, operating in an aqueous environment under mild conditions. Five of the enzymes in the cascade were evolved to overcome specific limitations and improve compatibility with non-natural substrates. Galactose oxidase (GOase) catalyzed the oxidative desymmetrization of the starting material **62**, while pantothenate kinase (PanK) enabled selective phosphorylation. Deoxyribose-5-phosphate aldolase (DERA) facilitated a diastereoselective aldol addition with acetaldehyde. Phosphopentomutase (PPM) then effected phosphate migration, and purine nucleoside phosphorylase (PNP) completed the glycosylation step using an adenine derivative (**65**) as nucleophile. Four not evolved auxiliary enzymes were incorporated to balance the reversible steps and facilitate cofactor recycling (Figure 14).^144^

**Figure 14:**
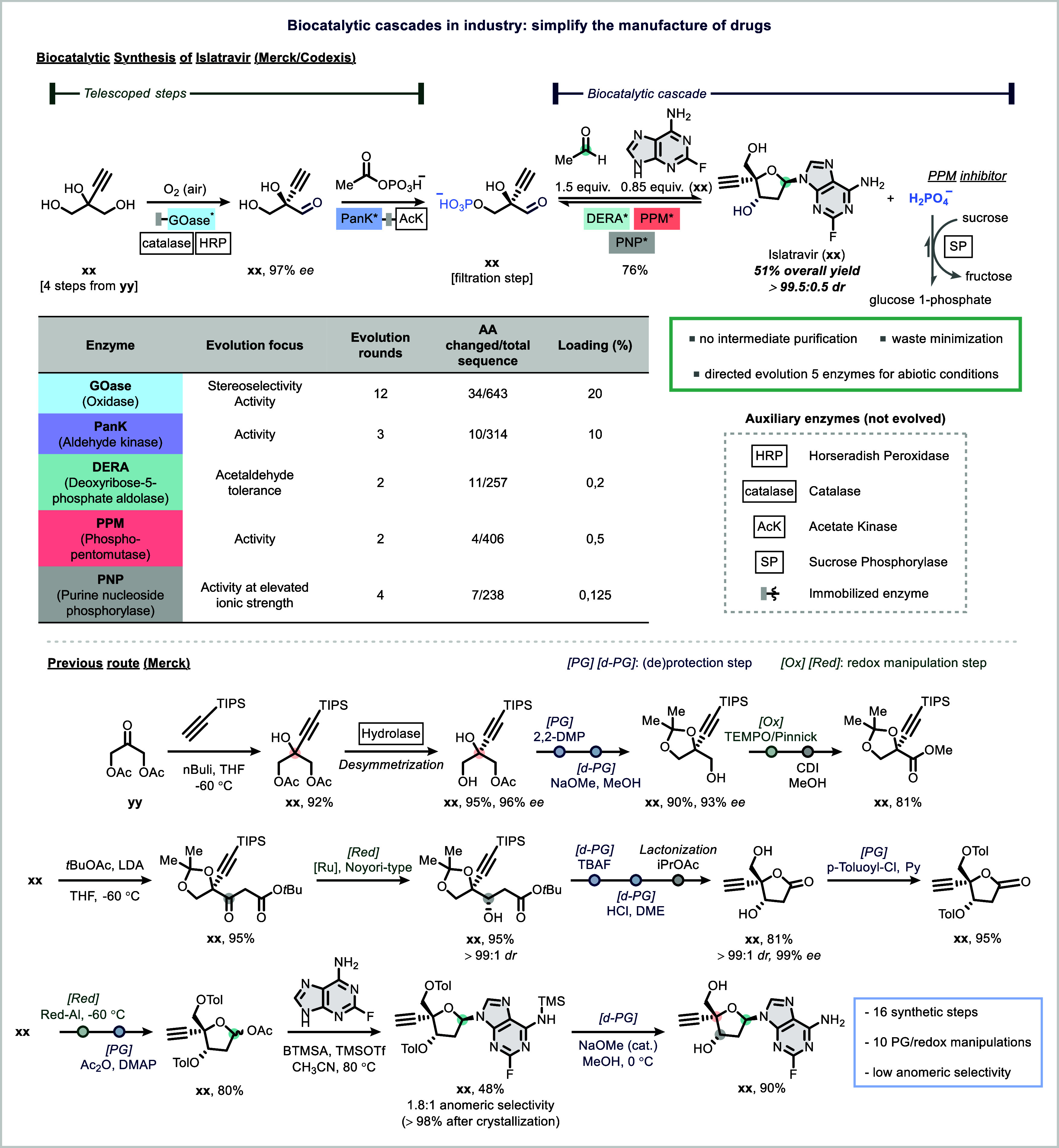
Application of engineered biocatalytic cascades to streamline the synthesis of Islatravir (**66**).

Directed evolution played a critical role in resolving key bottlenecks.^130^ For example, the tolerance of DERA to acetaldehyde was significantly improved, and both PPM and PNP were engineered to accept non-natural substrates with higher efficiency. Remarkably, the entire cascade was executed as a single-pot process without intermediate isolation, delivering a streamlined three-step synthesis with an overall yield of 51 and >95% product purity (**66**). This represented a dramatic improvement over Merck’s prior synthetic route, which required 16 synthetic steps, suffered from poor atom economy, and involved labor-intensive protecting group strategies and redox interconversions.^145^ By contrast, the enzymatic cascade offered a selective, efficient, and environmentally benign route to islatravir (**66**),^146^ underscoring the potential of engineered biocatalysts for the stereoselective and sustainable synthesis of complex pharmaceutical synthesis at scale.^127^

Similar to the advantages observed in heterogeneous catalysis, enzyme immobilization on polymeric resins offers significant benefits for industrial biocatalytic processes.^146,147^ Immobilization enables the repeated use of enzymes, reducing the overall cost of these valuable catalysts. Additionally, immobilized enzymes exhibit enhanced compatibility with a wide range of organic solvents; such conditions would typically lead to rapid denaturation of free enzymes.^148^ Beyond solvent tolerance, immobilization confers improved thermal and operational stability, enabling complete recyclability of the catalyst over multiple cycles. From a process development standpoint, these features translate into substantial reductions in enzyme loading, eliminate the need for aqueous buffers and strict pH control, and greatly simplify the isolation of water-soluble products.^149^ Collectively, these attributes align well with the pharmaceutical industry’s demands for productivity, robustness, and sustainability.^127^

Enzyme immobilization was used by scientists at Merck, who explored the reversible transamination of the biorenewable solvent Cyrene (**79**), converting its ketone functionality to a key amine intermediate (**80**) under aqueous buffer conditions.^150^ This transformation achieved high yield and satisfactory diastereoselectivity (Figure 15A). However, early development faced several practical challenges, including the need for constant pH adjustment, extended reaction times, high enzyme loadings, and a labor-intensive separation of the water-soluble product from the enzyme.^151^ To overcome these obstacles, the team employed a dual strategy of directed evolution and enzyme immobilization. The evolved transaminase variant ATA-492 was immobilized on ECR8415 resin, enabling the reaction to proceed in 2-MeTHF, a green, industrially preferred organic solvent. This optimized setup allowed for higher substrate concentrations, reduced the reaction time from 20 to 7 h, and facilitated easier product isolation (Figure 15B).^151^

**Figure 15:**
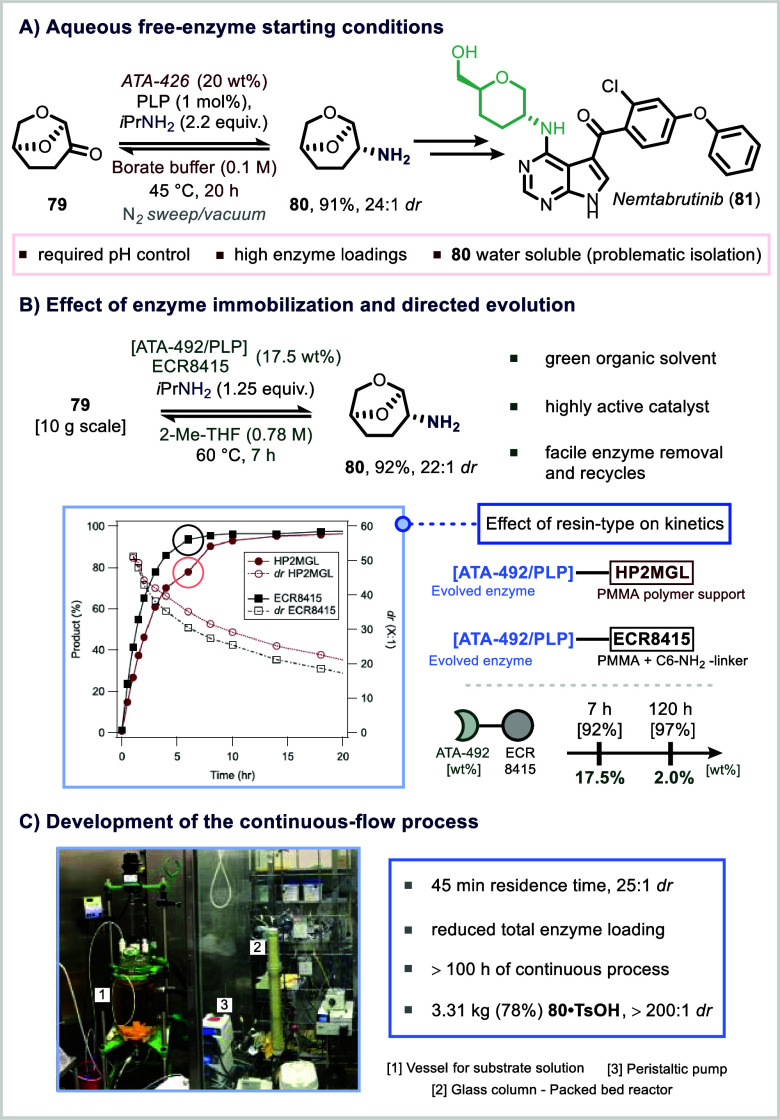
Immobilization of transaminase enzymes on resin. Reproduced from ref 152 with permission from American Chemical Society.

The selection of an appropriate resin support proved critical for reaction efficiency. Among several materials evaluated, ECR8415, an alkylamine-functionalized resin, delivered superior performance due to its selective adsorption of ATA-492, along with favorable properties such as larger pore size and an extended amine linker. These characteristics contributed to a 38% increase in the initial reaction rate compared to standard resins. Moreover, enzyme loading could be significantly reduced to as low as 2%, without compromising product yield or diastereoselectivity, though this extended the reaction time to 120 h (Figure 15B).^151^

Building on these findings, Merck successfully translated the immobilized transaminase system into a packed-bed flow reactor, enabling continuous production.^152^ This setup facilitated real-time optimization of parameters such as residence time, temperature, and substrate concentration, and mitigated enzyme inhibition through continuous product removal.^148,153,154^ Following rigorous process development, the system was scaled to produce over 3 kg of the amine intermediate (**80**) as its tosylate salt. Impressively, the reactor ran for over 100 h of continuous operation with no detectable loss in enzymatic activity (Figure 15C).^152^

A distinct advancement in the field of biocatalysis for organic synthesis lies in the strategic exploitation of enzyme promiscuity, the inherent ability of enzymes to catalyze non-native reactions with low but detectable activity, often due to mechanistic similarities with their natural functions.^155,156^ Several research groups have successfully harnessed this latent reactivity and, through directed evolution, transformed initially weakly active enzymes into efficient biocatalysts capable of promoting new-to-nature reactions, including carbene^157,158^ and nitrene^159,160^ transfer processes.^161^

Researchers have repurposed cofactor-dependent enzymes as photobiocatalysts capable of engaging in single-electron transfer (SET) reactions under visible-light irradiation.^162,163^ These systems mimic the reactivity of traditional photocatalysts while operating under benign aqueous conditions. However, even these advances remain fundamentally anchored in reactivities that preexist in nature and still rely on evolutionary optimization.

Despite the explosion of available sequence data from genome mining,^164^ the number of enzymes that are both mechanistically well-characterized and readily applicable to synthetic chemistry remains relatively small. This limitation narrows the scope of transformations accessible through current biocatalysis when compared to the vast diversity enabled by classical synthetic methods.^165^ Critically, for many valuable synthetic transformations, no known natural enzyme exists to serve as a starting point for directed evolution. This reflects an underlying structural constraint: the chemical functionality of enzymes is inherently restricted by the 20 canonical amino acids, limiting their ability to engage in broader catalytic chemistries.^142,165,166^

To overcome these challenges, recent efforts have turned to de novo enzyme design. Computational strategies, such as the theozyme model, which calculates theoretical active sites that stabilize specific transition states, have enabled the rational design of enzyme-like scaffolds with entirely novel functions.^167–169^ These designed proteins can, in principle, perform transformations inaccessible to native enzymes.

In parallel, alternative strategies have emerged to further expand the chemical space of enzyme catalysis. One promising approach is the creation of artificial metalloenzymes, where noble metal cofactors are embedded into protein scaffolds, enabling reactivities typically observed only in transition-metal catalysis.^141^ Another is the site-specific incorporation of noncanonical amino acids (nCAAs) into enzyme structures. These abiotic residues introduce new chemical functionalities that can act as catalytic centers, opening the door to reactions that natural enzymes cannot mediate.^142^

Advances in genetic code expansion now allow for the precise incorporation of nCAAs into strategically selected positions within protein hosts, particularly those with favorable structural features such as hydrophobic pockets or noncovalent binding regions.^140,142^ These modified proteins can then be subjected to directed evolution to enhance their activity, selectivity, and stability (Figure 16A). In this way, a growing class of engineered artificial enzymes is emerging, capable of bridging the mechanistic divide between biocatalysis and traditional small-molecule catalysis, and expanding the toolkit available for complex molecule construction.^143,170^

**Figure 16:**
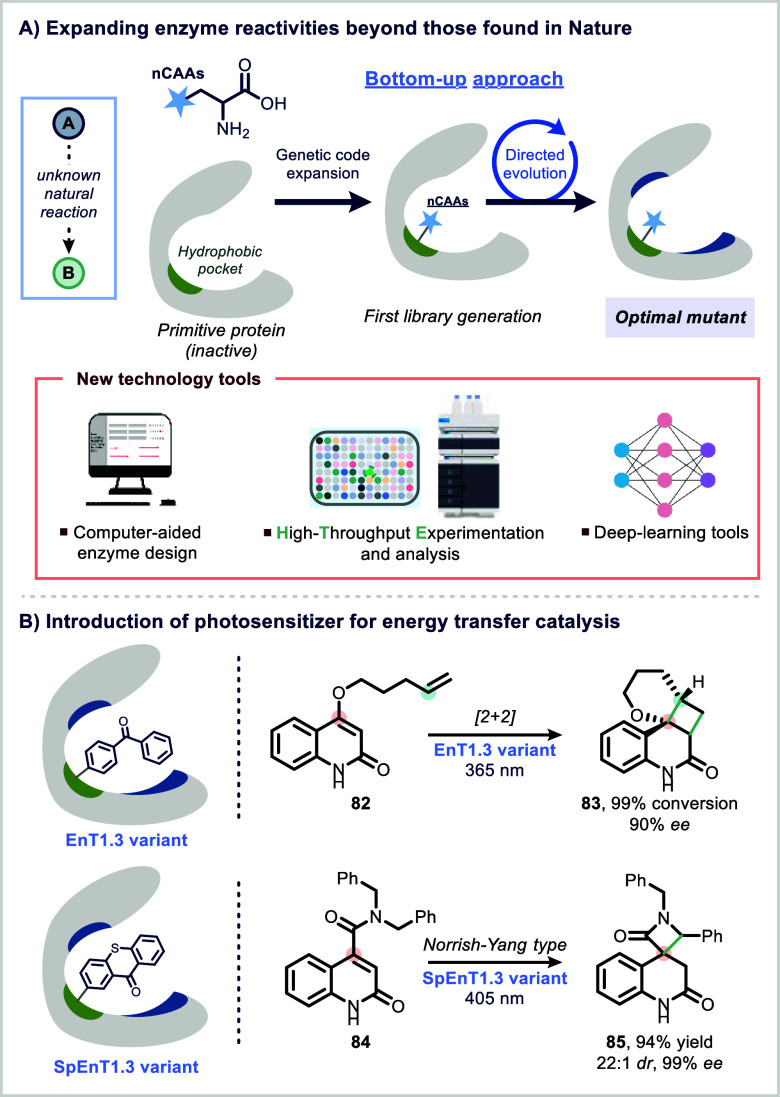
Non-natural enzymes via ncAA incorporation.

To further optimize these bottom-up approaches, researchers are increasingly turning to ultrahigh-throughput screening and advanced computational tools, including deep learning, to enhance catalytic performance and predict productive variants (Figure 16A). These innovations enable the rapid identification and refinement of enzyme candidates, greatly accelerating the design cycle and facilitating the development of artificial enzymes capable of catalyzing non-natural reactions.^171^

A promising application of this approach has been the design of artificial photoenzymes through the incorporation of noncanonical amino acids (nCAAs).^142^ Leveraging genetic code expansion, researchers have site-specifically embedded photocatalysts into protein skeletons by introducing nCAAs bearing inexpensive and well-characterized photosensitizers (Figure 16B).^172–174^ This modular strategy allows the photocatalyst to initiate light-driven transformations, while the protein environment provides a chiral framework that enables stereoselective control.

Green and co-workers used this concept to develop photoenzymes by incorporating classic photosensitizers, such as benzophenone and thioxanthone, into a computationally designed Diels–Alderase scaffold (DA_20_00). The resulting enzymes, **EnT1.3** and **SpEnT1.3**, catalyzed stereoselective energy transfer (EnT) reactions (Figure 16B).^173,175^ These engineered photoenzymes were applied to promote either intramolecular [2 + 2] cycloadditions or formal C–H insertion reactions via a Norrish–Yang-type mechanism under aerobic conditions. The transformations furnished complex bicyclic (**83**) and spirocyclic (**85**) products with high levels of enantioselectivity, showcasing the power of integrating photochemical and enzymatic principles within a single catalytic entity (Figure 16B).

Although still in its early stages, this approach, embedding abiotic catalytic functions via nCAAs, represents a highly promising strategy for enabling new classes of stereoselective transformations that lie beyond the reach of natural enzymes. That said, current applications remain largely confined to academic laboratories. To enable broader industrial adoption, there is a pressing need to streamline the design–optimization–testing cycle, reduce time and resource investment, and extend the applicability of these tailored biocatalysts to synthetically useful, process-relevant targets.^171^

## Mechanochemistry

Solvents are integral to the execution of chemical reactions, influencing not only yields and selectivity (regio-, chemo-, and stereoselectivity), but also the stabilization of reactive intermediates. Additionally, solvents serve critical functions in process safety, particularly for managing heat in exothermic reactions and avoiding thermal runaway. However, from a large-scale manufacturing perspective, solvent-related parameters such as toxicity, flammability, explosiveness, and waste generation must be carefully considered.^176^ Moreover, many sensitive reactions demand solvent pretreatment (e.g., degassing and drying), adding complexity and operational burden to the process.

Mechanochemistry has emerged as a powerful and sustainable alternative to traditional solvent-based synthesis.^177^ Recognized by IUPAC as one of the Top 10 Innovations in Chemistry in 2019,^178^ this green enabling technology allows for the execution of chemical transformations in the absence of bulk solvents or with minimal amounts, typically less than 1 μL/mg, a mode known as liquid-assisted grinding (LAG).^179,180^ Through mechanical energy via grinding, milling, or shearing, chemical reactions can be driven in the solid state, reducing environmental impact and simplifying setups. Critical parameters such as milling frequency, ball size, jar volume, milling time, and temperature significantly influence on reaction outcomes and must be carefully optimized for each transformation.^181^

An illustrative application of mechanochemistry is in the activation of zerovalent metals, which are widely employed in organic synthesis.^182,183^ These metals are essential for reactions such as direct metalation of alkyl halides to form organometallic intermediates, as well as reductants in cross-electrophile couplings and Birch-type reductions. However, the reactivity of zerovalent metals can be significantly compromised by surface oxidation, variations in particle size, and differences in physical form.^183^ Conventional activation methods typically involve chemical additives, such as iodine, 1,2-dibromoethane, or trimethylsilyl chloride, in solvent-based systems under strictly anhydrous and inert conditions (Figure 17, top left).^184^ These procedures are often labor-intensive, operator-dependent, and difficult to scale reliably. In contrast, mechanochemical activation via ball milling provides a more efficient, practical, and sustainable alternative. The mechanical force exerted during milling facilitates surface abrasion and crushing, effectively removing oxide layers and reducing particle size, thereby exposing the reactive metal core.^183^ As a result, this solid-state activation process is faster than traditional solution-based methods and eliminates the need for air- and moisture-sensitive solvents or elaborate reaction setups. (Figure 17, top right).^182,185^

**Figure 17:**
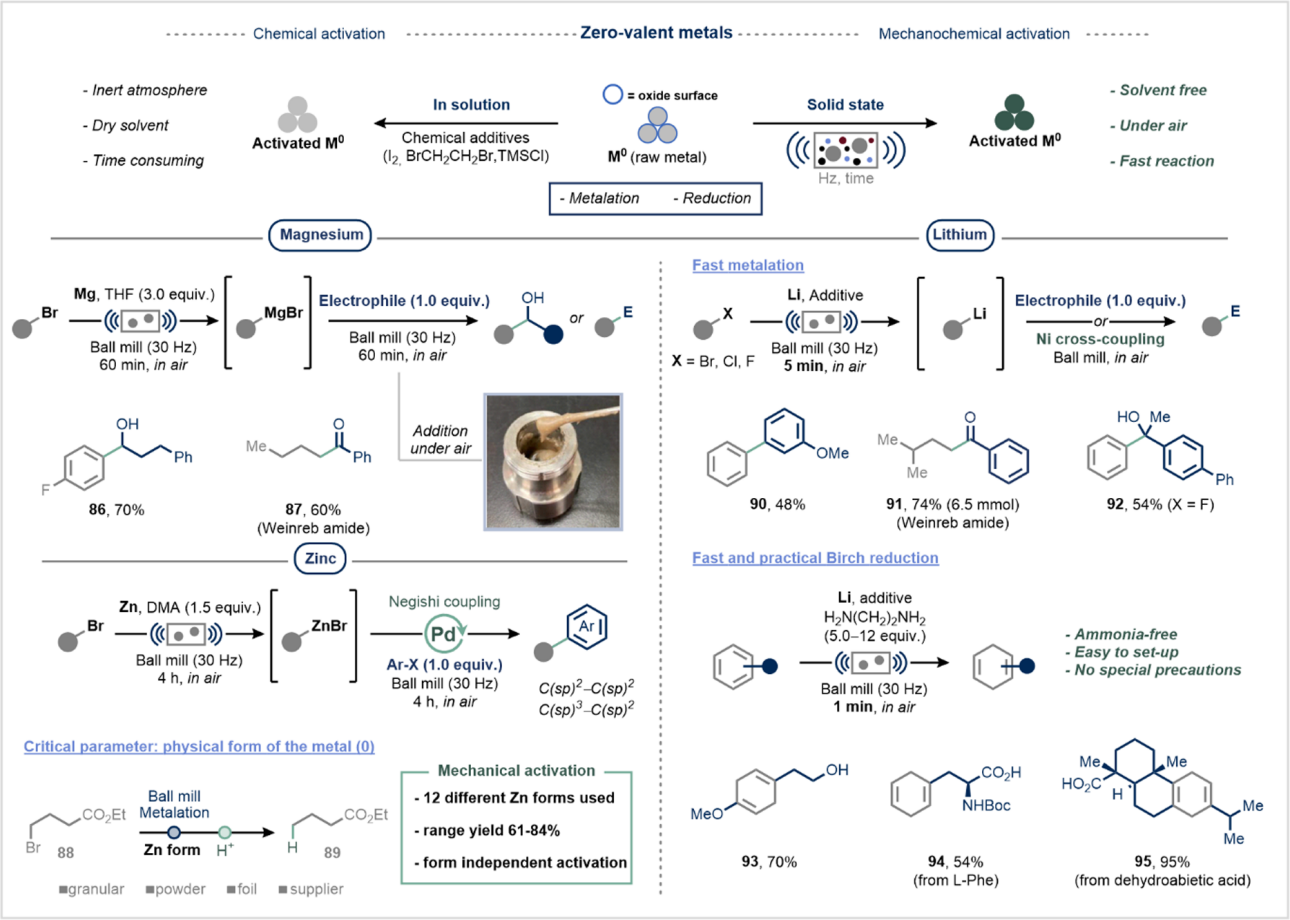
Mechanochemistry promotes the activation of zerovalent metals (Mg, Zn, Li): applications to the direct metalation of alkyl halides and Birch-type reduction. Reproduced from ref 186 with permission from Springer Nature.

The Ito group described in detail the solid-state activation of zerovalent metals, introducing a mechanochemical method for generating Grignard reagents under ambient conditions (Figure 17, Magnesium).^186^ In this approach, magnesium turnings, an aryl or alkyl bromide, and tetrahydrofuran (THF) as a liquid-assisted grinding (LAG) agent were placed in a stainless-steel jar and subjected to ball milling for 60 min. After milling, the jar was opened to air, and the electrophile was added, followed by a second grinding cycle to complete the transformation.

In a complementary study, the Browne group developed a solvent-free mechanochemical protocol for the activation of zinc metal, enabling the air-stable synthesis of organozinc reagents without requiring classical chemical activators (Figure 17, Zinc).^187^ This methodology was seamlessly integrated into a one-pot palladium-catalyzed Negishi cross-coupling, delivering both C­(sp^2^)–C­(sp^2^) and C­(sp^2^)–C­(sp^3^) products with broad functional group tolerance.^187^ Recognizing the variability of commercial zinc sources, the team evaluated 12 distinct forms of zinc and found that mechanochemical activation was effective across all tested forms, thereby enhancing reproducibility and simplifying access to organozinc reagents (Figure 17, Zinc).

Lithium is another key metal in organic synthesis, valued for its strong reducing power (*E* ^0^ = −3.04 V vs SHE), and is widely used in processes such as Birch reductions and the preparation of organolithium reagents.^188^ However, lithium’s high reactivity with air and moisture presents significant safety risks at scale.^189^ Ball milling offers a safer and more practical method for lithium activation under ambient conditions. The Ito group demonstrated a rapid (5 min) mechanochemical metalation strategy for synthesizing aryl and alkyl organolithium compounds, which were successfully transformed into downstream products via electrophilic trapping or nickel-catalyzed reactions (Figure 17, Lithium top).^190^

Notably, the protocol enabled the direct generation of aryl lithium species from otherwise unreactive aryl fluorides (**92**) and was scalable to 6.5 mmol (**91**). This platform was further adapted into an ammonia-free Birch reduction, completed in just 1 min (Figure 17, Lithium bottom).^191^ This user-friendly protocol efficiently reduced natural products to their corresponding dienes in high yield (**93**–**95**), demonstrating the potential of mechanochemistry to modernize classic transformations.

As a practical safety note, finely ground metal powders generated via ball milling can be pyrophoric when exposed to air, posing a fire hazard during isolation and handling.^183^

In photocatalysis, solvent choice is critical for stabilizing both excited states and charged intermediates.^192^ However, commonly used polar aprotic solvents pose safety hazards at scale and require rigorous oxygen exclusion to prevent quenching of excited triplet states.^193^ In response, recent studies have explored the integration of photocatalysis with mechanochemistry to reduce solvent use and conduct reactions under air.^193,194^ While promising, these approaches currently face challenges related to reactor design and scalability.^195^

An exciting development in this area is the emergence of mechanoredox catalysis, a mechanochemical alternative to photocatalysis that offers operational simplicity.^181,196^ The Ito group demonstrated the use of commercially available BaTiO_3_ nanoparticles as piezoelectric materials in ball milling. When subjected to mechanical stress, these particles generate localized electric potentials that trigger single-electron transfer (SET) processes, initiating radical chemistry in a manner analogous to the oxidative quenching step in photocatalytic cycles (Figure 18).^197^ This technology provides a solvent-minimized, scalable pathway to radical transformations without the need for light, photosensitizers, or inert conditions.^196,198^

**Figure 18:**
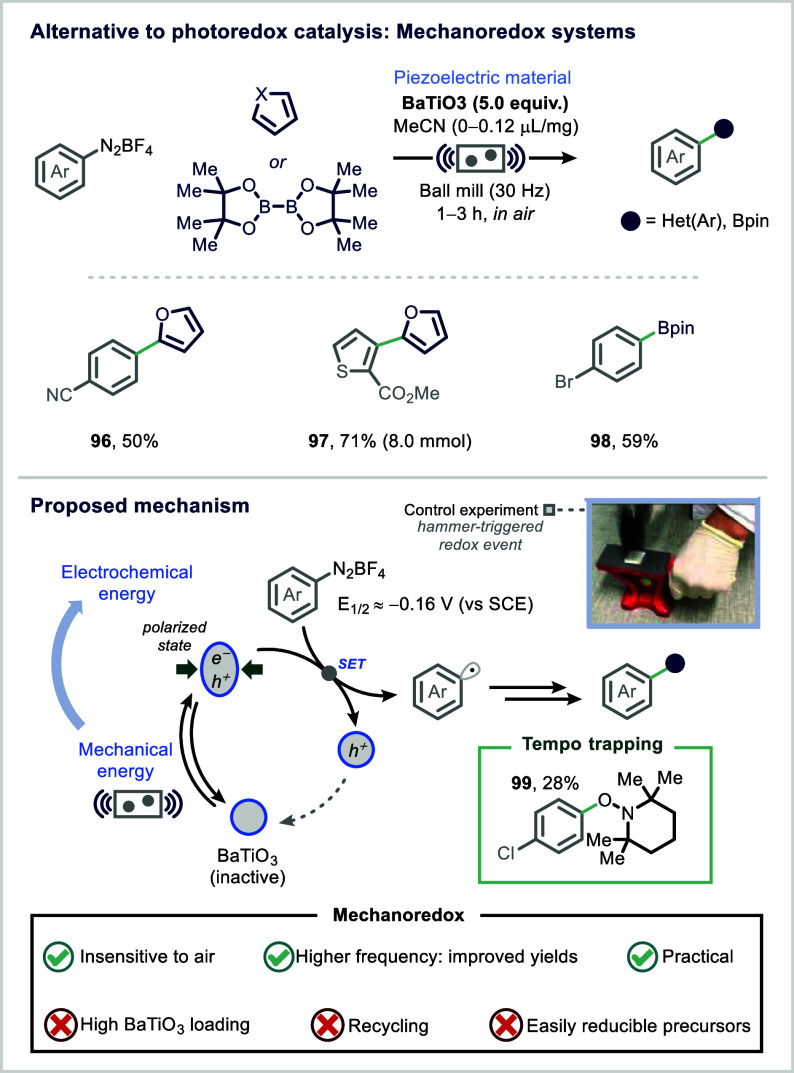
Mechanoredox ball milling. Reproduced from ref 197 with permission from The American Association for the Advancement of Science.

The electrochemical potential generated by the polarized state of BaTiO_3_ under mechanical stress was harnessed to facilitate single-electron transfer (SET) to aryl diazonium salts (*E*_1_/_2_ = −0.16 V vs SCE), generating aryl radicals under solvent-free, ambient conditions.^197,199^ These aryl radicals underwent subsequent addition to electron-rich heteroarenes or bis­(pinacolato)­diboron, affording C–H arylation or borylation products in moderate to good yields (27–80%). Mechanistic studies confirmed the radical nature of the transformation. Control experiments showed no product formation in the absence of the piezoelectric material, provided enhanced yields at increased milling frequencies, and displayed inhibition of product formation upon addition of the radical scavenger TEMPO (**99**). These findings strongly support a mechanoredox radical pathway driven by the piezoelectric effect (Figure 18).

Despite its promise, several limitations remain. The protocol currently requires high loadings of BaTiO_3_ (5 equiv), and the piezoelectric material can only be recycled for up to three cycles before significant loss of activity is observed. Furthermore, the method is presently restricted to substrates with low reduction potentials, limiting broader application. Looking forward, advancements in the design of next-generation piezoelectric materials with improved durability and broader redox windows will be crucial for expanding the scope and practicality of mechanoredox catalysis.

Mechanochemistry has gained recognition as a promising strategy for sustainable chemical manufacturing, particularly due to its solvent-minimized nature, which dramatically reduces *E*-factors and waste in large-scale synthesis.^185^ However, the upscaling of mechanochemical reactions remains a significant challenge. Limitations include the small volume capacity of traditional ball mills, insufficient temperature control, and the lack of standardized, scalable protocols.^176^ To address these issues, twin screw extrusion (TSE), a continuous processing technology widely used in polymer and food industries, has been adapted for continuous mechanochemical organic synthesis.^200–202^ In TSE, solid reactants are fed into a barrel containing two intermeshing screws that convey, mix, and react the materials along the screw axis in a modular and controlled fashion (Figure 19). This approach offers several advantages over batch milling, including enhanced scalability, reproducibility, and process intensification.

**Figure 19:**
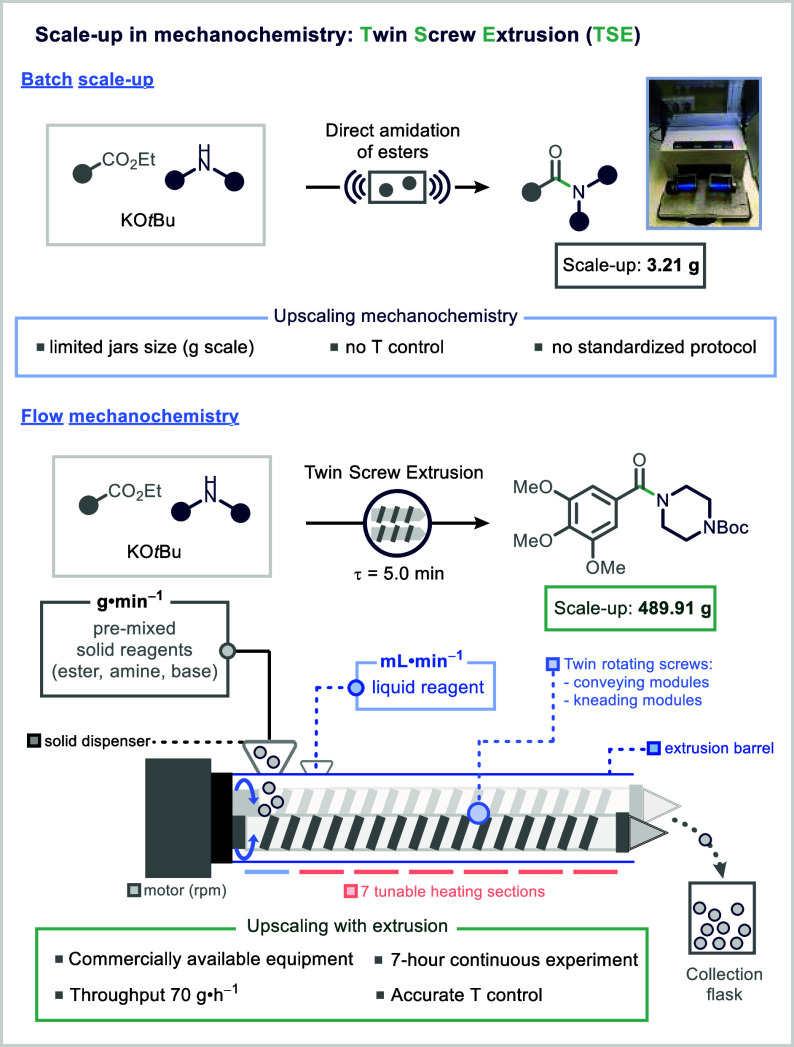
Upscaling of mechanochemistry in continuous flow. Reproduced from ref 204 with permission from Wiley-VCH GmbH.

A notable pharmaceutical application of TSE is the direct amidation of esters, eliminating the need for stoichiometric activating agents in the synthesis of secondary and tertiary amides.^203^ The Browne group successfully translated this transformation from a batch ball-milling protocol to a solvent-free continuous extrusion process, achieving a 100-fold scale-up.^204,205^ In a 7-h continuous run, approximately 500 g of the desired amide were isolated (Figure 19).^205^ Process optimization required careful adjustment of multiple operational parameters, including temperature, feed rate, screw speed, screw configuration, and the physical properties of the starting materials.

## Outlook and Conclusions

The landscape of synthetic organic chemistry is undergoing a profound transformation, driven by the integration of enabling technologies that are steadily shifting the focus from purely manual craftsmanship to a more data-rich, automated, and design-driven discipline. While round-bottom flasks and batch reactors remain central to chemical education and research, the increasing accessibility of flow reactors, electrochemical setups, photocatalytic systems, and self-driving laboratories offers chemists the opportunity to radically rethink how reactions are conducted, scaled, and optimized.

As highlighted throughout this perspective, these technologies are no longer fringe alternatives but robust platforms that address concrete limitations of classical synthetic approaches, whether by improving reaction stereoselectivity, simplifying purification, minimizing hazardous conditions, or enabling reactions that were previously considered impractical. The success of technologies, such as directed evolution in biocatalysis and twin-screw extrusion in mechanochemistry, further illustrates the tangible benefits of embracing a more interdisciplinary and sustainable mindset for process scale manufacturing.

Nonetheless, broader adoption will depend on continued efforts to democratize these tools through open-source hardware,^206^ user-friendly software, modular platforms, and revised educational curricula. Chemists must become fluent not only in the language of molecules but also in that of engineering, data science, biotechnology, enzyme engineering and even automation. Training the next generation to navigate this expanded toolkit will be crucial to ensure that technology serves not as a barrier but as a catalyst for innovation.

Looking forward, the convergence of synthetic chemistry with digital infrastructure, including machine learning, cloud-connected automation, and high-throughput experimentation, promises to unlock truly autonomous discovery cycles. Self-driving laboratories are just the beginning of a broader shift where hypotheses are tested and refined by algorithms, materials are made on-demand, and reactivity landscapes are mapped faster and with higher precision. In this emerging paradigm, human creativity and machine accuracy will work together to accelerate progress across medicinal chemistry, materials science, and beyond.

Our hope is that this perspective encourages researchers to critically engage with enabling technologies, not as luxury tools for niche problems but as essential components for a more agile, sustainable, and innovative synthetic future.
